# P-Texture Effect on the Fatigue Crack Propagation Resistance in an Al-Cu-Mg Alloy Bearing a Small Amount of Silver

**DOI:** 10.3390/ma11122481

**Published:** 2018-12-06

**Authors:** Yangcheng Hu, Zhiyi Liu, Qi Zhao, Song Bai, Fei Liu

**Affiliations:** 1 Key Laboratory of Nonferrous Metal Materials Science and Engineering, Ministry of Education, Central South University, Changsha 410083, China; csuhuyangcheng@163.com (Y.H.); zhaoqi2012@163.com (Q.Z.); baisongmse@163.com (S.B.); mutong1606@163.com (F.L.); 2 School of Material Science and Engineering, Central South University, Changsha 410083, China

**Keywords:** Al-Cu-Mg-Ag alloy, P-texture, roughness induced crack closure, fatigue crack propagation resistance, damage tolerance

## Abstract

P-texture effect on the fatigue crack propagation (FCP) resistance in an Al-Cu-Mg alloy containing a small amount of Ag, is investigated by X-ray diffraction (XRD), scanning electron microscopy (SEM), transmission electron microscopy (TEM) and electron back scattering diffraction (EBSD). Results shows that the high intensity P-texture sheet has lower $\sigma_{0.2}/\sigma_{b}$, lower FCP rate and higher damage tolerance than random texture sheet. Fracture analysis indicates that the striations spacing of high intensity P-texture sheet is much smaller than that of random texture sheet and it has a rougher fatigue fracture surface, which causes a significant roughness induced crack closure (RICC) effect. The calculation results manifest that high intensity P-texture sheet possesses a higher crack closure level reaching 0.73 as compared to random texture sheet (only 0.25). The statistical analysis results reveal the P-grains have large twist angle of 105–170° and tilt angle of 5–60° with neighboring grains, which is similar to Goss-grains. This is the fundamental reason that P-texture sheet has the same FCP resistance and induces fatigue crack deflection as Goss-texture sheet. Additionally, the most {111} slipping planes of P-grains are distributed in the range of 30–50° deviating from transverse direction of the sheet. This results in more {111} slipping planes to participate in cyclic plastic deformation, which is beneficial to reduce fatigue damage accumulation and improve the damage tolerance of Al-Cu-Mg-Ag alloy.

## 1. Introduction

Previous researches indicated that the fatigue properties of polycrystalline materials were not only controlled by co-clusters and second-phase particles, but also related to grain structures, such as grain size, boundary and orientation. Some researchers [1,2,3] have highlighted the influence of Cu-Mg and Mg-Ag co-clusters on the fatigue crack propagation (FCP) of Al-Cu-Mg alloys. Their results revealed large co-clusters were beneficial for improving FCP resistance. The effect of second-phase particles, that is Ω, θ’, T_1_ phases [2,4,5] on fatigue behavior in aluminum alloys has also been examined. These shearable particles gave rise to crack deflection and reduced FCP rates. However, coarse Si- and Fe-rich intermetallic particles had adverse effect on fatigue properties as they often acted as the crack initiation sites and formed micro cracks [6]. Grain size was a vital structural factor affecting fatigue behavior. It was well known that grain refinement could enhance FCP resistance [7,8,9]. Some investigations about the influence of grain boundaries on FCP resistance in aluminum alloys were discussed. The results showed that high-angle grain boundaries were deleterious to fatigue properties [10]. However, twin boundary was considered to be a special type of coherent high-angle grain boundary with low interfacial energy [11]. It possessed higher FCP resistance than high-angle grain boundaries [12]. Of course, the FCP resistance can be improved by reducing the width of precipitation free zone (PFZ) at grain boundaries [13].

In recent years, several attempts have been made to find out the relationship between FCP behavior and grain orientation. Zhai et al. [14] proposed a crystallographic model to predict the process of crack deflection at the grain boundaries. They believed that the twist angle and tilt angle at grain boundaries were the key factors in controlling the short fatigue crack growth. Later, the influence of individual grain orientation on fatigue crack growth behavior in Al-Li and Al-Cu alloys was investigated [15]. Results indicated Goss-grains possessed higher resistance to FCP across grain boundaries than Brass-grains. Liu et al. [16] confirmed that Goss-texture combined with a part of Cube-texture induced great fatigue crack deflections due to the large twist angle or tilt angle component boundaries with neighboring grains. But Brass-texture showed little resistance to FCP. Recently, Li et al. [17] found individual P-grain could induce fatigue crack deflection in Al-Cu-Mg alloy. However, there are still numerous debates on the work of Li about P-grains effect on FCP behavior in Al-Cu-Mg alloys. The reasons are chiefly as follows: the first one is the relationship between fatigue behavior and grain orientation that is twist angle and tilt angle at grain boundary, is not well clarified by quantitative analysis. The second is the available evidence is still not sufficient to illustrate the effect of P -texture, because only one P-grain is observed during the investigation of fatigue crack growth. Therefore, it still remains a great challenge to explore the P-texture effect on FCP resistance and clarify its mechanism. In addition, P-texture {110} 〈122〉 in Al-Cu-Mg alloys is not a common texture component like Cube, Goss or Brass. Jung et al. [18] used crystal plasticity theory and strain energy release maximization theory to study the nucleation mechanism of P-orientations near a coarse precipitate of a plane strain compressed Al alloy. Their results showed that the nucleation of P-texture was primarily possible due to stable P-orientations and near P-orientations formed during plane strain compression, which acted as nuclei that recrystallize into P-orientations. Huang et al. [19,20] demonstrated the importance of concurrent precipitation on P-texture. Their work revealed that concurrent precipitation suppressed nucleation at grain boundaries, which allowed the growth of P texture components which possessed special orientation relationships with the surrounding deformed matrix. However, the intensity of P-texture obtained in Al-Cu-Mg alloys is relatively low. There is still no work explaining how to strengthen the P-texture intensity of Al-Cu-Mg alloys. Thus, how to obtain high intensity P-texture in Al-Cu-Mg alloys requires intensive research.

Many studies have shown that different metallic chemical constituents in aluminum alloys had essential influence on stacking fault energy (SFE). The magnitude of SFE governs the ease of dislocation cross-slip, which plays an important role in controlling the formation of rolling textures [21,22,23]. Schulthess et al. [21] demonstrated the addition of Ag in range of 0 to 30 at.% could increase the SFE of Al-Cu-Mg-Ag alloy as compared to Al-Cu-Mg alloy. Different SFE resulted in different texture evolution during hot rolling process. Furthermore, the hot rolling texture affected the recrystallization behaviors and thus had a vital influence on the formation of recrystallized textures [24]. In order to obtain high intensity P-texture, a small amount of Ag is added into Al-Cu-Mg alloy to enhance the SFE, which further changes the dislocation slipping mechanism during hot rolling and affects the formation and evolution of recrystallization texture during annealing.

Accordingly, the present work proposes a method to obtain high intensity P-texture in Al-Cu-Mg alloys by adding a small amount of Ag with suitable annealing parameters. The objective of the present work is to reveal the effect and mechanism of P-texture on FCP behavior with a purpose to establish the relationship between FCP resistance and texture.

## 2. Materials and Methods

The material used in this work was an Al-Cu-Mg-Ag alloy sheet (50 mm thick). Its nominal composition was 4.00% Cu, 1.45% Mg, 0.54% Ag, 0.52% Mn, 0.04% Ti, 0.04% Fe, 0.02% Si (in wt.%) and the remainder Al. The sheet was hot rolled to 5 mm after being homogenized at 495 °C for 48 h. Subsequently heat treatment, water quenching, solution treatment and natural aging were applied to adjust grain orientation. Based on the different annealing temperature, the specimens were named as samples A and B. The detailed processes of these sheets were present in Table 1.

**Figure 1:**
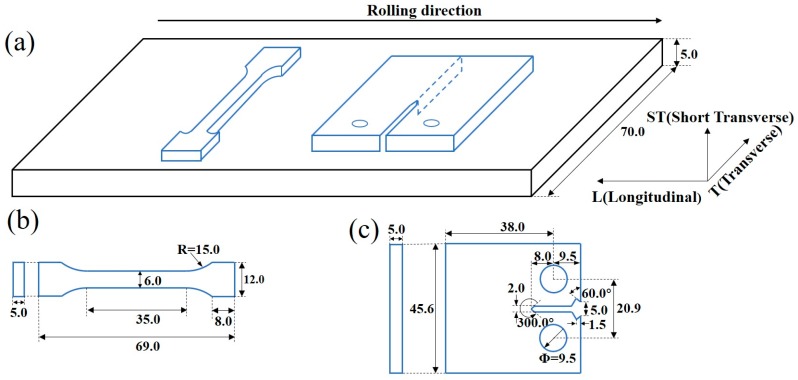
Schematic diagrams showing the specimens for tensile and fatigue crack propagation (FCP) tests: (**a**) the orientation and location of the specimens taken from the rolling plates, the geometry and dimensions of the (**b**) tensile and (**c**) FCP specimens. (unit in mm).

The geometry and dimensions of the tensile specimen and FCP specimen were illustrated in Figure 1. The tensile tests were carried out on an Instron testing machine with a cross-head speed of 2 mm/min. FCP tests were performed on an MTS-810 machine using compact tension (CT) specimens, which were taken from the natural aged sheet in L-T orientation with a size of 45.6 mm × 38.0 mm × 5.0 mm (length, width and thickness). Tests were conducted in air at room temperature with a sinusoidal cyclic constant loading frequency of 10 Hz at a stress radio (R = σ_min_/σ_max_) of 0.1.

Transmission electron microscopy (TEM) analysis was conducted on a TECNAI G^2^ 20 microscope (FEI, Hillsboro, OR, USA) with an operating voltage of 200 kV. The sample used for TEM observation was a 3 mm thin disk. It was twin-jet electropolished in an 70% methanol and 30% nitric acid at approximately −20 °C. The macro-texture was performed on a Bruker D8 Discover X-ray diffractor (Bruker, Karlsruhe, Germany). The (111), (200) and (220) pole figures were measured by the Schulz back reflection method using Cu Kα radiation. The orientation distribution functions (ODFs) were calculated from the pole figures using the series expansion method. Electron back scattering diffraction (EBSD) technique was used to obtain grain orientation on both sides of long cracks. Specimens were prepared by conventional mechanical grinding and subsequently electro-polishing. EBSD measurements were carried out on a Sirion 200 field emission gun scanning electron microscope (SEM, FEI, Hillsboro, OR, USA) with an accelerating voltage of 20 kV. Fatigue fracture surfaces were analyzed by a FEI Quanta 200 SEM operating at 20 kV. Simultaneously, a three-dimensional profiles of fracture surface roughness were obtained by using Hirox Kh7700 (Hirox, Tokyo, Japan). 

## 3. Results

### 3.1. Mechanical Properties and Textures

**Figure 2:**
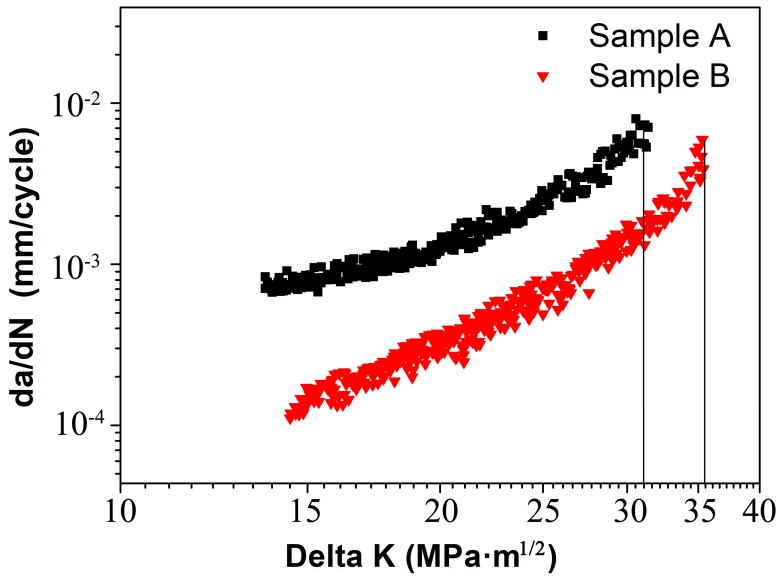
FCP rates, da/dN, as a function of Delta K for samples A and B.

The results of tensile tests are given in Table 2. The variation of FCP rates, da/dN, with stress intensity range, ΔK for samples A and B are shown in Figure 2. Sample B exhibits lower FCP rate than sample A. For instance, at ΔK = 15 MPa·m^1/2^, FCP rates of samples A and B were respectively 8.15 × 10^−4^ and 1.54 × 10^−4^ mm/cycle. Likewise, in the final stage (ΔK = 30 MPa·m^1/2^), FCP rates of samples A and B are respectively up to 6.25 × 10^−3^ and 1.59 × 10^−3^ mm/cycle. It is clear that sample B presents higher FCP resistance. Besides, sample B possesses higher damage tolerance (35.5 MPa·m^1/2^) than sample A (31 MPa·m^1/2^).

**Figure 3:**
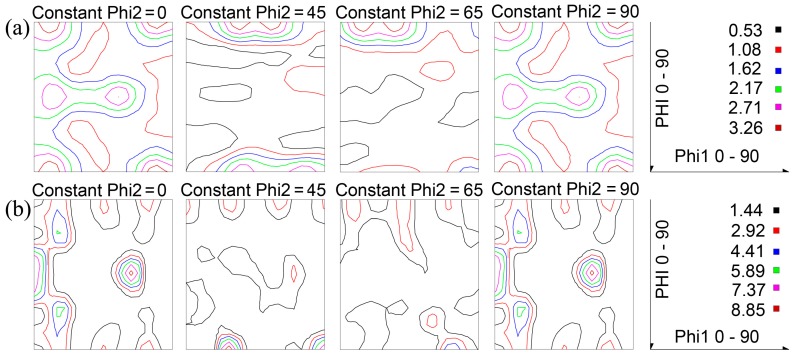
Texture measurement results showing orientation distribution functions (ODFs) section for sample A (**a**) and sample B (**b**).

Figure 3 demonstrates the texture measurement of samples A and B. The texture of sample A is random texture. By contrast, the texture in sample B is predominated by high intensity P-texture (8.85) and Goss-texture (7.37). In previous studies, the intensity of P-texture in the Al-Cu-Mg alloys without the addition of Ag was only about 4 [17]. However, the present work obtains high intensity P-texture with strength level of 8.85 in Al-Cu-Mg alloys by adding a small amount of Ag and then optimizing annealing parameters. Obviously, the intensity of P-texture has greatly improved. 

The comparison of FCP rates and macro-texture (see Figure 2 and Figure 3) suggests that high intensity of P- and Goss-textures are responsible for the low FCP rate of sample B. The random texture, however, results in the high FCP rate of sample A. Obviously, the grain orientation plays an important role in governing FCP behavior of two samples, although other microstructural factors, that is, grain size and second-phase particles, may affect the fatigue properties.

### 3.2. Fatigue Crack Propagation Morphology

**Figure 4:**
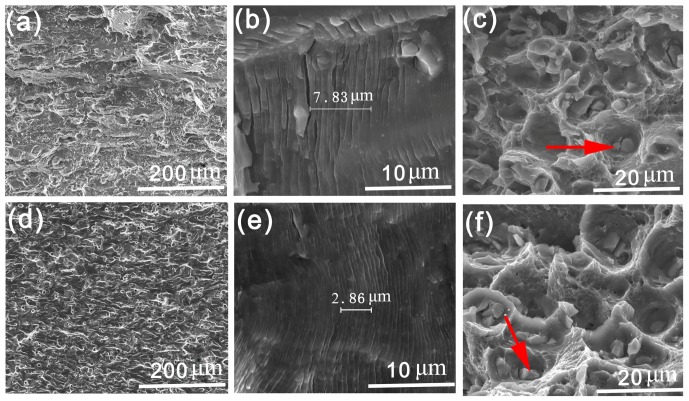
Microstructure of fatigue fracture surfaces in different stages for sample A (**a**–**c**) and sample B (**d**–**f**).

Figure 4 shows the fatigue fracture surfaces in different stages of samples A and B. In Figure 4a,d (ΔK = 15 MPa·m^1/2^), since the shear deformation plays a dominant role in this stage, the typical features are characterized by beach marks that are roughly paralleled to the FCP direction. Moreover, fatigue plateaus connecting with each other by tear ridges are also detected in both samples. Interestingly, sample B exhibits more microscopic voids as compare to sample A. Srivatsan et al. [25] revealed that these microscopic voids were mainly due to the localized plasticity deformation around the coarse second-phase particles. Figure 4b,e show the fatigue fractures of various samples in Paris regime (ΔK = 23 MPa·m^1/2^). The values in Figure 4b,e are the spacing of ten fatigue striations that we measured with the Nano Measurer software. Sample B possesses finer fatigue striations than sample A. When cracks extend to the high ΔK regime (ΔK = 30 MPa·m^1/2^), the fatigue fracture surfaces are mainly composed of dispersed dimples. It also should be noted that there are some particles in the center of the dimples and some of them are broken (as seen in Figure 3c,f). Energy spectrum analysis indicates the atomic ratios (Al:Cu:Mg) of the detected particles (see red arrows in Figure 3c,f) are 57.13:25.56:12.34 and 60.72:22.71:13.81, respectively. These second particles are probably S (Al_2_CuMg) phase. 

**Figure 5:**
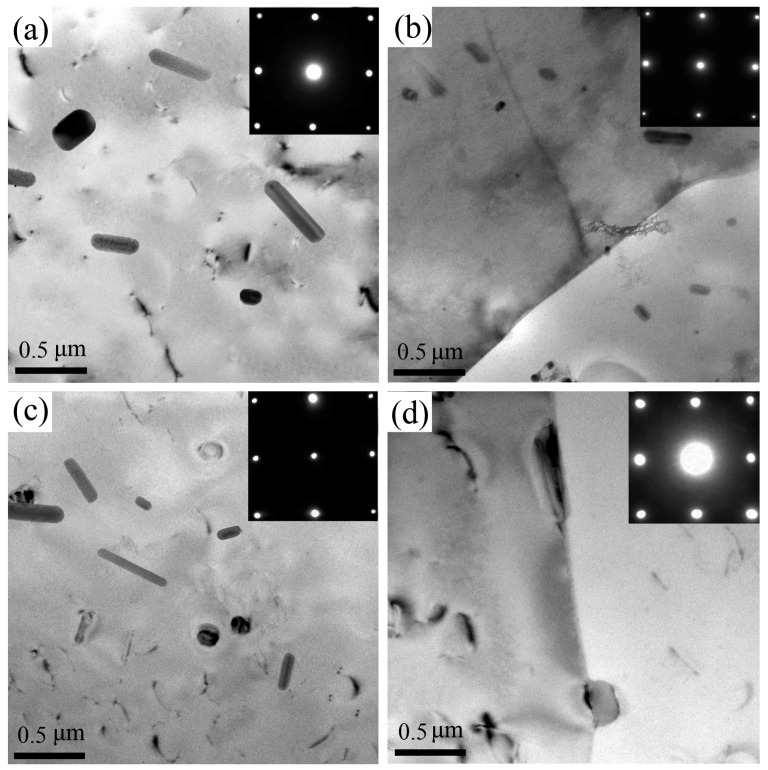
TEM bright field micrographs and corresponding SAED patterns (<100>α zone axis) of sample A (**a**,**b**) and sample B (**c**,**d**).

The microstructures of samples A and B are given in Figure 5. As revealed from the selected area electron diffraction (SEAD) taken near a <100>α zone axis, no distinct precipitates are detected in both natural aged samples, but some large T-phase particles (Al_20_Cu_2_Mn_3_) [26,27] and a few dislocations are observed. Moreover, from Figure 5b,d, we can see that samples A and B do not form precipitation free zone (PFZ) at grain boundary under natural aging.

**Figure 6:**
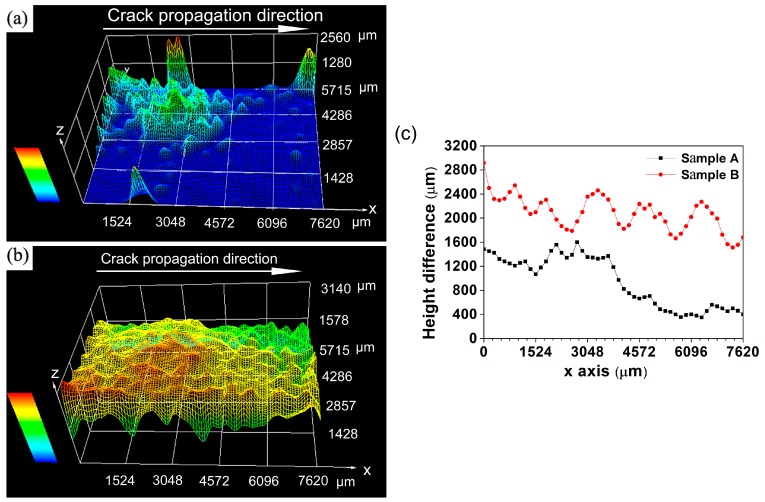
Three-dimensional fatigue fracture surface of sample A (**a**), B (**b**) and height dif0ference between top point and bottom point of samples A and B along the crack propagation direction (**c**).

Figure 6 illustrates the three-dimensional fatigue fracture surfaces of samples A and B. In order to gain a more thorough understanding of the effect of roughness induced crack closure (RICC) on the FCP rate, the height difference of fatigue fracture surface is calculated via KH7700 3DViewer (see Figure 6c, Hirox, Tokyo, Japan). Apparently, sample B possesses a relative rougher fatigue fracture surface than sample A. Besides, the height difference of fatigue fracture surface of sample B is much higher than that of sample A.

**Figure 7:**
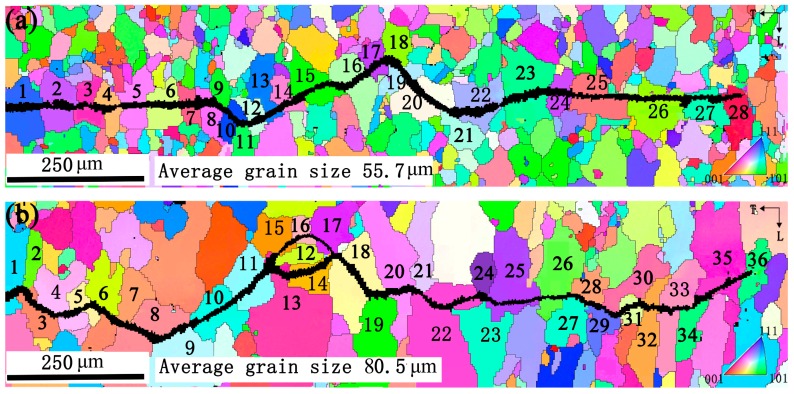
EBSD results showing fatigue crack propagating path for samples A (**a**) and B (**b**).

The Miller indices of grains near fatigue cracks in Figure 7a,b are given in Table 3 and Table 4 respectively. From Figure 7a and Table 3, the orientations of grains leading to crack deflections are close to P (grains 9, 16, 18, 21 and 23) and Goss (grain 11) in sample A. Similarly, the orientations of the marked grains in sample A are Cube (grains 3, 7 and 25), Brass (grain 1), L (grains 10, 13 and 15), R (grains 8, 12, 14, 20 and 27) and Copper (grains 2, 5, 17 and 24), respectively. Likewise, from Figure 7b and Table 4, it can be seen that P-grains 6, 12, 21, 23, 28 and 29 induce dominant deflections in sample B. Fatigue crack deflections also occur at Goss-grains 2, 9, 19 and 34. Other deflections are also observed when fatigue crack propagated through grains 3 and 16 (see Figure 7b), which are close to Cube. However, it is important to point out that grains 32 and 33 in sample B can be penetrated directly by crack with little deflection (see Figure 7b). Their orientations are also close to Cube. Besides, the orientation of grains 10, 11, 26 and 31 are close to Brass, grains 4, 15 and 18 are close to H, grains 17, 24 and 25 are close to Copper. It becomes evident that P-grains and Goss-grains possess larger FCP resistance than other oriented-grains.

## 4. Discussion

### 4.1. Effect of Grain Orientation on FCP

Earlier work has shown that grain refinement is an effective way to enhance FCP resistance [7,8,9]. The quantity of grain boundaries increased with the decreasing grain size, which could impede fatigue crack growth and lead to higher FCP resistance. However, this conclusion does not appear to be supported by the present results. From Figure 2 and Figure 7, sample B shows a lower FCP rate than sample A even though its grain size is larger than that of sample A. Apparently, the smaller grain size does not always lead to a lower FCP rate. Accordingly, there is another factor governing FCP resistance of this alloy.

As viewed from the microstructures of samples A and B (see Figure 5), the dislocation density of samples A and B has no distinct difference, the number and the size of T-phase are similar. Moreover, samples A and B do not form PFZ at grain boundary. As for the co-cluster, previous investigations revealed that the size of co-clusters has a great relationship with the aging state and alloy composition [1,2,3]. Therefore, the size of Cu-Mg and Mg-Ag co-clusters are not much different between samples A and B because they have the same aging state and alloy composition. Obviously, there are more important factors affecting the FCP rates of samples A and B.

The distinct microstructural difference between samples A and B is the grain orientation, as seen in Figure 3. Sample B with high intensity P-texture (8.85) and Goss-texture (7.37) has better fatigue performance. Sample A with random texture exhibits a higher FCP rate (see Figure 2). Based on the above discussion, grain size, second-phase particles, PFZ and co-clusters all present little effect on FCP. It is reasonable to conjecture that different texture components in two samples are responsible for different FCP rates. However, what is the reason for the different texture components induce different FCP rates? Careful examination of crack path and the orientation of grains near fatigue crack in Figure 7, Table 3 and Table 4 reveal the fatigue crack is obviously deflected or obstructed when passing by P-grains, that is grains 9, 16, 18, 21 and 23 in Figure 7a and grains 6, 12, 21, 23, 28 and 29 in Figure 7b. Similarly, Goss-grains can also induce crack deflections, such as grain 11 in Figure 7a and grains 2, 9, 19 and 34 in Figure 7b. On the contrary, no crack deflections are observed when fatigue crack penetrates through Copper-, Brass- and R-grains. As for Cube-grains, different Cube-grains presented different FCP resistance. What’s the mechanism for the different FCP behaviors presented by these different oriented-grains? Previous investigations by Liu [16] revealed that Goss-grains combined with a part of Cube-grains induced great fatigue crack deflections due to the large twist angle or tilt angle component boundaries with neighboring grains. However, there are still numerous debates on the previous work about grain orientation (especially the P-grains) effect on FCP behavior in Al-Cu-Mg alloys. The relationship between fatigue behavior and grain orientation, that is, twist angle and tilt angle at grain boundary, is not well clarified by quantitative analysis in previous studies [16,17].

**Figure 8:**
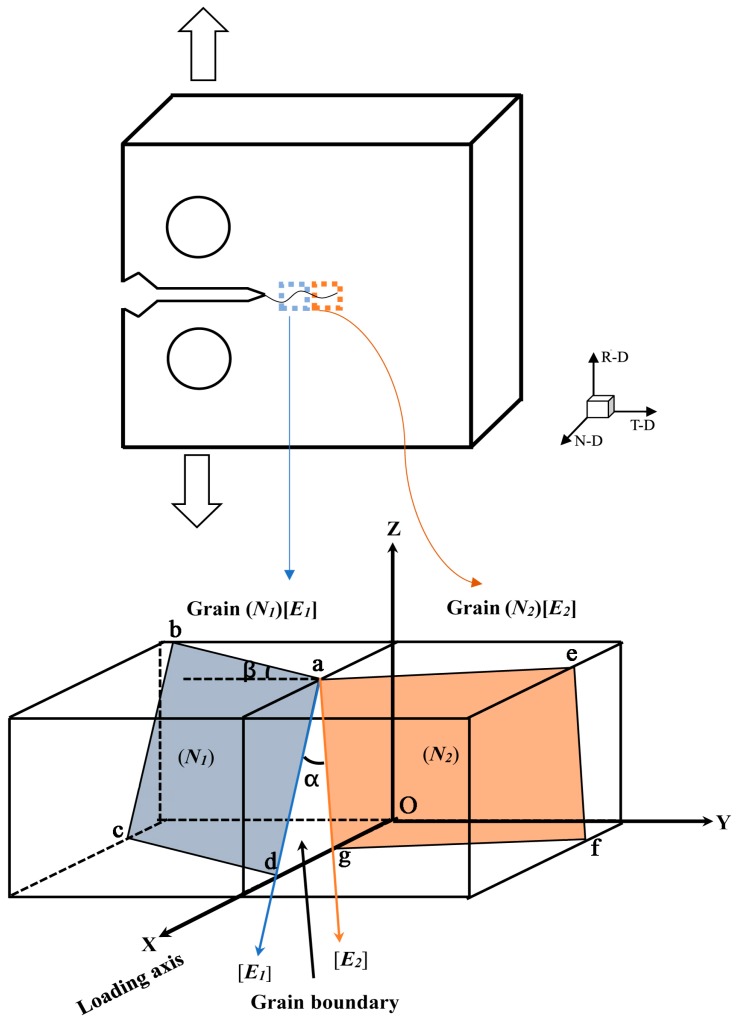
Schematic diagram showing a crystallographic mechanism for fatigue crack growth in Paris regime.

As analyzed above, precise quantitative is required to study the influence of grain orientations on FCP resistance. Wu et al. [28] proposed a crystallographic model to calculate the twist angle and tilt angle but this model was only applicable for near-threshold regime. Generally, in Paris regime, the crack tip plastic deformation is distinctly different from that in near-threshold regime. Thus, in order to accurately study the influence of grain orientations on FCP resistance in fatigue stage II, a modified crystallographic model for evaluate the twist angle and tilt angle component boundaries is established in the present work. Figure 8 illustrates the orientation relationship of two adjacent grains. Actually, the blue crystal plane (***N*_1_**) and crystal direction [***E*_1_**] of the marked grain 1 should be kept parallel to the orange one, because they are parallel to the surface of the EBSD specimen. Hence, in order to keep parallel with the orange crystal plane, the blue direction [***E*_1_**] of grain 1 should firstly rotate around the normal unit vector of grain boundary (define as ***Z***) until the crystal direction of [***E*_1_**] is parallel to [***E*_2_**] and then the rotated blue crystal plane (***N*_1_’**) also must be rotated again along the ag line until the two crystal planes are parallel. By this rotation, the grain boundary characteristic such as twist angle and tilt angle can be determined quantitatively. The twist angle can be expressed by:

***α* = *arccos*(*E*_1_·*E*_2_)** (1)

As for ***Z***, which is the unit vector of grain boundary normal, is given by:

***Z* = (*E*_1_ × *E*_2_)/|*E*_1_·*E*_2_|** (2)

where ***E*_1_** and ***E*_2_** are the crystal direction of grains 1 and 2, respectively. In order to characterize tilt angle, one needs to know the crystal plane (***N*_1_’**) obtained by the ***N*_1_** plane rotating α angle around ***Z***. The rotated crystal plane (***N*1’**) can be calculated using the equation:

***N1*’ = *Z*·(*Z*·*N*_1_) (1 − *cosα*) + (*Z* × *N*_1_)·*sinα* + *N*_1_·*cosα*** (3)

Thus, the tilt angle of grain 1 with grain 2 can be described as:

***β* = *arccos*(*N*_1_’·*N*_2_).** (4)

**Figure 9:**
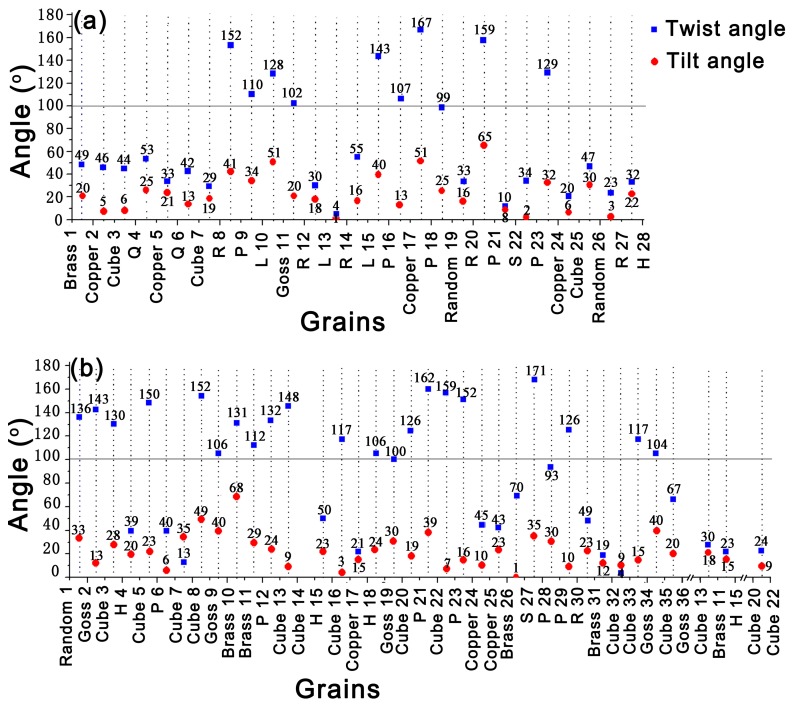
The twist angle and tilt angle between neighboring grains for samples A (**a**) and B (**b**) (Note: the perpendicular dotted line between the neighboring grains represents the grain boundary.).

The calculated twist angle ***α*** and tilt angle ***β*** are shown in Figure 9. It is apparent that fatigue crack in sample A deflects severely by P-grains (see grains 9, 16, 18, 21 and 23 in Figure 7a). The twist angles of R-grain 8 and P-grain 9, L-grain 15 and P-grain 16, Copper-grain 17 and P-grain 18, R-grain 20 and P-grain 21, Copper-grain 24 and P-grain 23 are respectively 152°, 143°, 167°, 159°and 129°. The large twist angle induces great crack deflection or retardation at those grain boundaries, which is responsible for the enhanced FCP resistance. Similarly, for sample B, P-grains 6, 12, 21, 23, 28 and 29 are also detected to possess large twist angle of 112–171° with adjacent grains and crack deflections occur at those grain boundaries accordingly. For instance, a great crack deflection is observed when fatigue crack develops across the boundary of Cube-grain 5 and P-grain 6, as the twist angle of P-grain 6 with Cube-grain 5 is extremely large reaching 150°. Fatigue crack propagates along the boundary of P-grain 21 and penetrates through Cube-grains 20 and 22 directly. This is because the twist angle between P-grain 21 with Cube-grain 20 is 126° and that of P-grain 21 with Cube-grain 22 is 162°. However, the twist angle of Cube-grains 20 and 22 is only 24°. Likewise, the main fatigue crack is obstructed by P-grain 23 when the crack tries to from Cube-grain 22 into P-grain 23, then the crack retraces its way back to Cube-grain 22, as the twist angle between P-grain 23 and Cube-grain 22 is 159°. Besides, it should be note that the main fatigue crack is obstructed by P-grain 12, subsequently divides into two branches and propagates through Cube-grain 13 and H-grain 15, respectively. This is because the twist angle between P-grain 12 and Brass-grain 11 is 112°. However, Brass-grain 11 is observed to have a small twist angle with Cube-grain 13 (about 30°) and H-grain 15 (about 23°), which contribute to little resistance to FCP. Therefore, fatigue crack propagates through Cube-grain 13 and H-grain 15 but arrests by P-grain 12. Based on these results, it can be concluded that the twist angle component boundary plays a major role in controlling crack deflection and retardation. Obviously, P-grains possessing large twist angle with neighboring grains present high FCP resistance. 

Goss-grains 11 in sample A and 2, 9, 19 and 34 in sample B also possess large twist angle of 100–152° with neighboring grains, which induce crack deflections at those grain boundaries. This conclusion was compatible with that of Liu et al. [16] who found out that Goss-grains had large twist angle component boundaries with adjacent grains in Al-Cu-Mg alloy. Fatigue crack penetrates through Cube-grains 3, 7 and 25 (see Figure 7a), grains 32 and 33 (see Figure 7b) directly, which attribute to the small twist angle component boundary of 4–46°with neighboring Q-, Copper- or Brass-grains. However, some Cube-grains (see grains 3 and 16 in Figure 7b) are observed to have large twist angle with neighboring H-grain (130°) or Copper-grain (117°) and then give rise to the relatively great crack deflections. This suggested that FCP resistance of Cube-grains depend on its twist angle with adjacent grains. The twist angle of Copper-grains with neighboring Q-, R- or H-grains are relatively small (about 21–53°), which present low FCP resistance. Similarly, R-grains 8, 12, 14 and 27 in sample A possessing small twist angle of 4–55° with neighboring Cube-, L- or H-grains, result in no crack deflections when fatigue crack developed through these grains. Brass-grains show little resistance to FCP even though they have a large twist angle with neighboring grains (see grains 10 and 11, grains 26 and 27 in Figure 7b) as fatigue damage is easily accumulated in these grains [16].

**Figure 10:**
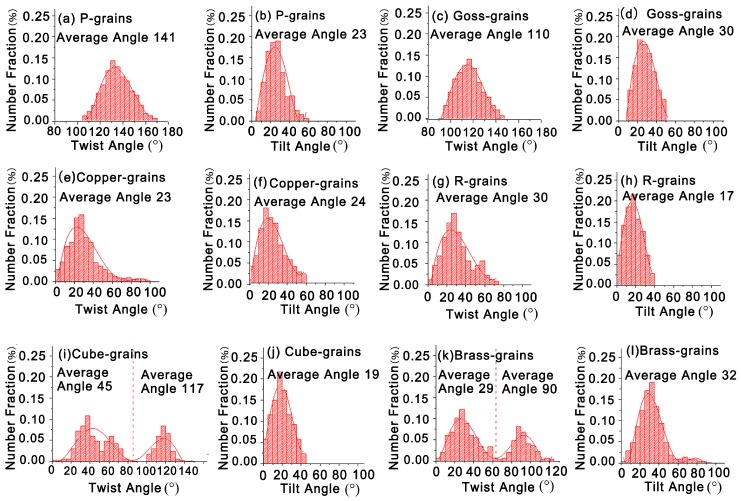
The twist angle and tilt angle distribution of P-grains with its neighboring grains (**a**,**b**); Goss-grains with its neighboring grains (**c**,**d**); Copper-grains with its neighboring grains (**e**,**f**); R-grains with its neighboring grains (**g**,**h**); Cube-grains with its neighboring grains (**i**,**j**); Brass-grains with its neighboring grains (**k**,**l**).

It should be clear that the twist angle component boundary plays an important role in governing the process of crack deflection and retardation at the grain boundary in Al-Cu-Mg-Ag alloy. Figure 10 illustrates the twist and tilt angle distributions of some specific oriented-grains with its neighboring grains, which are calculated from all the grains shown in Figure 7. The statistical analysis results reveal that P-grains have large twist angle of 105–170° and tilt angle of 5–60° with neighboring grains. Likewise, Goss-grains possess a large twist angle of 90–150° and tilt angle of 10–50° with adjacent grains. Obviously, P-grains and Goss-grains have similar grain boundary structure with neighboring grains. Previous studies revealed that similar grain boundary structures have similar effects on FCP [16,17,28]. This result shows that P-grains have the similar mechanism and effect on FCP resistance as same as Goss-grains in Al-Cu-Mg alloy. Moreover, the large twist angle is responsible for the great crack deflection or retardation at those grain boundaries. This is the fundamental reason why P-texture sheet possesses large FCP resistance. Conversely, the grain boundaries of other grains such as Copper-, R-, Cube- and Brass-grains are completely different from those of P- or Goss-grains (see Figure 10). Copper-grains have relatively small twist angle of 0–90° and tilt angle of 0–60° with surrounding grains. Similarly, R-grains have small twist angle of 0–70° and tilt angle of 0–40° with neighboring grains. Besides, it should be emphasized that the twist angle distribution of Cube-grains with its neighboring presents two peaks (see Figure 10i), corresponding to large average twist angle of 117° and small average twist angle of 45°. This provides a reasonable interpretation of different FCP resistance caused by Cube-grains. Brass-grains show small FCP resistance even though some of them have large twist angle. This phenomenon is elucidated by the accumulation of fatigue damage [16].

**Figure 11:**
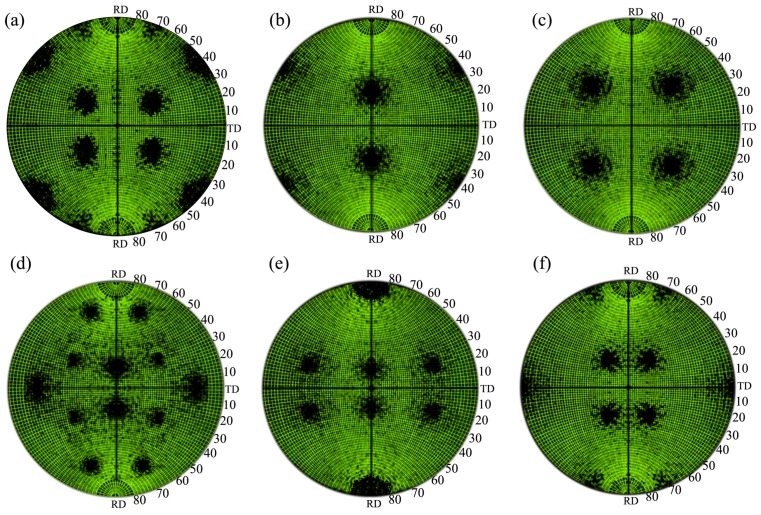
{111} pole figures of various standard textures: (**a**) P-texture, (**b**) Goss-texture, (**c**) Cube-texture, (**d**) R-texture, (**e**) Copper-texture and (**f**) Brass-texture.

As stated above, high intensity of P-texture induces large FCP resistance, which results in a lower FCP rate of Al-Cu-Mg alloy. This give a good explanation for low FCP rate of sample B. But why does sample B also have a higher damage tolerance than sample A? Figure 11 shows the {111} pole figures of various standard textures. Careful analysis of these pole figures indicates that the {111} slipping planes of P-grains are distributed in the range of 30–50° deviating from transverse direction (TD) of the sheet, which is similar to Goss- and Cube-grains. The {111} slipping planes of Goss-grains are distributed in the range of 25–45° and those of Cube-grains are distributed in the range of 25–55° (see Figure 11a–c). This suggests that the direction of {111} slipping planes of P-grains, Goss-grains and Cube-grains are closer to the maximum shear stress, resulting in more {111} slipping planes participate in cycle plastic deformation in these grains. This can improve dislocation slipping, reduce stress concentration and fatigue damage accumulation. Conversely, the {111} pole figure of the other oriented-grains have a distinct different {111} slipping plane distribution compared to P-, Goss- and Cube-grains. For instance, most {111} slipping planes of R-grains, Copper-grains and Brass-grains are distributed in 0–30°, 10–30° and 20–35° deviating from transverse direction (TD) of the sheet, respectively. Besides, a part of {111} slipping planes are even parallel to the rolling direction (RD) of the sheet (see Figure 11e–f). This indicates the direction of {111} slipping planes of R-grains, Copper-grains and Brass-grains are far away to the maximum shear stress, leading to little {111} slipping planes participate in cycle plastic deformation in these grains. In this way, stress concentration and fatigue damage accumulation are easily occurred in these oriented grains. Obviously, under the same external stress, P-, Goss- and Cube-grains have more {111} slipping planes to participate in cyclic plastic deformation, which is beneficial to promote the reciprocating slip of dislocation near fatigue crack tip, reduce stress concentration and fatigue damage accumulation rate and improve damage tolerance of Al-Cu-Mg-Ag alloy. In present study, the texture of sample A is random texture. By contrast, the texture of sample B is predominated by high intensity P-texture and Goss-textures. Therefore, sample B presents a higher damage tolerance than sample A.

### 4.2. Effect of Texture on Crack Closure Behavior

**Figure 12:**
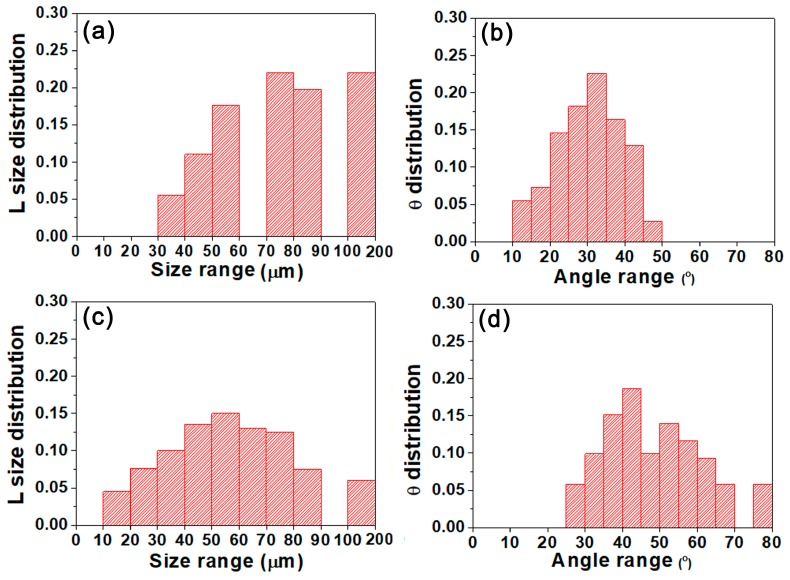
The distribution of L and θ for sample A (**a**,**b**) and sample B (**c**,**d**).

Previous work has identified crack deflection angle and asperity size are the main factors in determining RICC influence [29,30]. Their results suggested that increasing level of crack closure occurred with increasing crack deflection angle and asperity size. Recent work by Kamp et al. [31] has proposed an analytical model to estimate fatigue crack closure level. This approach uses K_cl_/K_Imax_ to express crack closure level:

(5) $$\frac{K_{\mathrm{cl}}}{K_{\mathrm{Imax}}}=\beta \frac{\sqrt{3\pi }K_{\mathrm{Imax}}\left(\left(1/2\right)+R-\left(1/2\right)R^{2}\right){\left(\sin\left(\theta /2\right)+\sin\left(3\theta /2\right)\right)}^{2}\sin2\theta }{16\sigma_{0.2}\left(3\cos\left(\theta /2\right)+\cos\left(3\theta /2\right)\right)}L\left(a^{*}\right)$$

with $L\left(a^{*}\right)=\frac{1}{\sqrt{a^{*}}}$, for $a^{*}>{\lambda r}_{p}$ and $L\left(a^{*}\right)=\frac{\sqrt{a^{*}}}{{\lambda r}_{p}}$, for $a^{*}\ll {\lambda r}_{p}$. Where K_Imax_ is the maximum mode I stress intensity factor. R, θ and $\sigma_{0.2}$ are load ratio, crack deflection angle and yield strength, respectively. a* is the deflection length (L) factor, which varies from 0 to L. FE modelling suggests a reasonable value for λ to be of the order of 0.4. The plastic zone size, $r_{p}$, can be calculated using Dugdale approximation: $r_{p}=\frac{1}{24\;\pi }\;\left(\frac{K_{\mathrm{Imax}}}{\sigma_{0.2}}\right)$ [32]. As for β, is a scaling factor approximately between 1 and 4. In this work, we take a* and β equal to L and 1, respectively. In order to use Equation (5), some geometrical parameters are required, such as deflection angle θ and deflection length L, which calculate from the real fatigue crack path shown in Figure 7. The distribution of L and θ parameters for samples A and B are presented in Figure 12. Moreover, the average of these parameters is used to calculate the closure level. Considering the crack closure behavior calculated by Equation (5) for samples A and B shown in Table 5, it can be seen that sample B has a higher closure level reaching 0.73 as compared to sample A (only 0.25). What is the reason for the different crack closure behavior of two samples? 

As stated above, sample B possesses high intensity P-texture. Conversely, the texture of sample A is random texture. From Section 4.1, it becomes evident that P-grains possessing large twist angle component boundary with neighboring grains are responsible for the enhanced deflection angle and fatigue fracture surface roughness (see Figure 6 and Figure 7). In fact, the average deflection angle of sample B is up to 47°. However, that of sample A is only 30° as shown in Table 5. Besides, careful examination of three-dimensional fatigue fracture surface in Figure 6 reveals that sample B possesses a rougher fracture surface and larger fatigue fracture height differences. However, sample A exhibits a smoother surface and lower fatigue fracture height differences. Accordingly, the asperity size of fatigue fracture surface in sample B is larger than that in sample A. Previous work documented that crack closure level increased with the raising of crack deflection angle and asperity size [29,30]. This view agreed with our observation. Sample B is found to have a higher fatigue crack deflection angle and larger asperity size, which in turn lead to increased RICC influence. Also, it has been reported that the fatigue behavior of aluminum alloys was affected by RICC [33]. Their results suggested that an increasing RICC level could enhance FCP resistance. As discussed above, sample B with high RICC level caused by P-texture presents higher FCP resistance as compared to sample A. This is another reason why P-texture can improve FCP resistance of the Al-Cu-Mg-Ag alloy.

## 5. Conclusions

(1) High intensity P-texture sheet has lower $\sigma_{0.2}/\sigma_{b}$, lower FCP rate and higher damage tolerance than random texture sheet.
(2) High intensity P-texture induces significant fatigue crack deflection and severe RICC effect up to 0.74, which are responsible for the large FCP resistance of Al-Cu-Mg-Ag alloy.
(3) P-grains possess large twist angle of 105–170° and tilt angle of 5–60° with neighboring grains, which is similar to Goss-grains. However, the grain boundaries of other grains such as Copper-, R-, Cube- and Brass-grains are distinct different from that of P- or Goss-grains. This is the fundamental reason that P-grains give rise to great fatigue crack deflection as Goss-grains.
(4) Similar to Goss-grains, the most {111} slipping planes of P-grains are distributed in the range of 30–50° deviating from transverse direction (TD) of the sheet, which is close to the direction of maximum shear stress. This is beneficial to promote the reciprocating slip of dislocation near fatigue crack tip, reduce stress concentration and fatigue damage accumulation rate and improve damage tolerance of Al-Cu-Mg-Ag alloy.

## Figures and Tables

**Table 1:** Experiment procedures of hot rolled Al-Cu-Mg-Ag alloy sheet.

Procedures	Experimental Parameters
I	Hot rolling at 450–460 °C + annealing at 320 °C/4 h + 490 °C/20 min + water quenching + natural aging (sample A)
II	Hot rolling at 450–460 °C + annealing at 420 °C/4 h + 490 °C/20 min + water quenching + natural aging (sample B)

**Table 2:** Tensile properties of Al-Cu-Mg-Ag alloy sheet.

Samples	Yield Strength (σ_0.2_) (MPa)	Tensile Strength (σ_b_) (MPa)	σ_0.2_/σ_b_	Elongation (%)
A	302	474	0.64	19.7
B	290	462	0.62	22.4

**Table 3:** The orientations of grains near fatigue crack in sample A.

Grain	Measured Indices	Simplified Indices	Measured Texture
1	(6 8 9)[21 9 −22]	Close to (111)[221]	Close to Brass
2	(6 −7 −13)[9 4 2]	Close to (112)(210)	Close to Copper
3	(5 4 19)[9 22 −7]	Close to (100)(321)	Close to Cube_ND_
4	(1 4 −13)[−2 7 2]	Close to (013)[311]	Close to Q
5	(11 −9 25)[−6 1 3]	Close to (112)[210]	Close to Copper
6	(0 4 9)[−26 −9 4]	Close to (013)[631]	Close to Q
7	(3 5 22)[−1 27 -6]	Close to (100)[100]	Close to Cube
8	(7 4 8)[−4 11 −2]	Close to (122)[412]	Close to R
9	(19 −1 21)[−6 −9 5]	Close to (011)[112]	Close to P
10	(13 12 15)[12 −13 0]	Close to (111)[110]	Close to L
11	(2 15 22)[−29 −2 4]	Close to (011)[100]	Close to Goss
12	(4 13 23)[14 −6 1]	Close to (124)[210]	Close to R
13	(16 12 17)[3 −4 0]	Close to (111)[110]	Close to L
14	(8 5 20)[15 −20 −1]	Close to (124)[110]	Close to R
15	(24 1 33)[29 −3 −21]	Close to (011)[110]	Close to L
16	(2 8 13)[−17 14 6]	Close to (011)[122]	Close to P
17	(11 12 20)[−20 20 −1]	Close to (112)[110]	Close to Copper
18	(3 11 19)[16 −13 5]	Close to (012)[122]	Close to P
19	(13 6 20)[14 −27 −1]	Close to (123)[210]	Random
20	(4 9 17)[2 1 −1]	Close to (124)[211]	Close to R
21	(5 0 8)[−16 9 10]	Close to (011)[122]	Close to P
22	(5 9 13)[21 10 −15]	Close to (123)[634]	Close to S
23	(1 6 7)[5 12 −11]	Close to (011)[221]	Close to P
24	(12 11 22)[−22 −2 13]	Close to (112)[210]	Close to Copper
25	(2 5 −23)[−10 27 5]	Close to (001)[310]	Close to Cube_ND_
26	(4 0 7 )[−21 1 12]	Close to (012)[012]	Random
27	(5 17 20)[−11 −5 7]	Close to (134)[211]	Close to R
28	(2 −2 25)[−1 −1 0]	Close to (001)(110)	Close to H

**Table 4:** The orientations of grains near fatigue crack in sample B.

Grain	Measured Indices	Simplified Indices	Measured Texture
1	(9 4 9)[1 9 −5]	Close to (122)[210]	Random
2	(5 0 8)[8 −28 −5]	Close to (011)[100]	Close to Goss
3	(1 6 27)[3 13 −3]	Close to (001)[100]	Close to Cube
4	(7 4 16)[−12 17 1]	Close to (001)[110]	Close to H
5	(2 7 15)[7 −2 0]	Close to (012)[100]	Close to Cube_RD_
6	(0 1 2)[−16 14 −7]	Close to (011)[122]	Close to P
7	(2 7 −27)[−24 3 1]	Close to (001)[100]	Close to Cube
8	(2 1 7)[−4 −27 5]	Close to (001)[100]	Close to Cube
9	(8 3 −12)[3 20 7]	Close to (011)[100]	Close to Goss
10	(6 17 20)[23 −14 9]	Close to (011)[211]	Close to Brass
11	(16 7 22)[−5 2 3]	Close to (011)[211]	Close to Brass
12	(11 1 23)[15 19 −8]	Close to (012)[221]	Close to P
13	( 3 1 14)[−9 −1 2]	Close to (001)[10 0]	Close to Cube
14	(−4 0 13)[ 26 1 8]	Close to (001)[310]	Close to Cube_ND_
15	(3 7 17)[−17 17 −4]	Close to (001)[110]	Close to H
16	(3 11 23)[4 1 −1]	Close to (012)[100]	Close to Cube_RD_
17	(1 1 2)[−4 6 −1]	Close to (112)[110]	Close to Copper
18	(7 10 −22)[−4 5 1]	Close to (012)[110]	Close to H
19	(0 3 4)[26 12 −9]	Close to (011)[100]	Close to Goss
20	(4 −2 7)[1 −12 −4]	Close to (123)[100]	Close to Cube_RD_
21	(1 4 6)[−18 21−11]	Close to (011)[122]	Close to P
22	(9 11 36)[ 13 −27 5]	Close to (001)[100]	Close to Cube
23	(3 12 17)[−10 11 −6]	Close to (011)[122]	Close to P
24	(7 6 11)[4 -23 10]	Close to (112)[012]	Close to Copper
25	(13 9 19)[5 −3 −2]	Close to (112)[111]	Close to Copper
26	(1 12 18)[6 10 −7]	Close to (011)[211]	Close to Brass
27	(1 3 3)[−21 19 −12]	Close to (133)[535]	Close to S
28	(2 12 19)[5 −4 2]	Close to (012)[122]	Close to P
29	(4 3 5)[−17 −9 19]	Close to (111)[122]	Close to P
30	(−5 3 18)[ 21 11 4]	Close to (126)(210)	Close to R
31	(12 3 22)[6 −2 −3]	Close to (012)(211)	Close to Brass
32	(3 1 11)[23 −3 −6]	Close to (013)[100]	Close to Cube_RD_
33	(4 2 11)[6 −1 −2]	Close to (013)[100]	Close to Cube_RD_
34	(5 1 6)[9 −21 4]	Close to (011)[100]	Close to Goss
35	(3 3 11)[51 4 −15]	Close to (001)[100]	Close to Cube
36	(14 3 17)[11 −23 −5]	Close to (011)[210]	Close to Goss

**Table 5:** The theoretical results of crack closure level for samples A and B.

Samples	K_Imax_ (MPa·m^1/2^)	σ_0.2_ (MPa)	L (μm)	λr*_p_* (μm)	θ (°)	K_cl_/K_Imax_
A	25.32	302	76	36	30	0.25
B	23.04	290	52	33	47	0.73
